# Antiviral Effect of Polyphenolic Substances in *Geranium wilfordii Maxim* against HSV-2 Infection Using *in vitro* and *in silico* Approaches

**DOI:** 10.1155/2022/7953728

**Published:** 2022-05-18

**Authors:** Hao Zhang, Zhen Li, Chaoqun Li, Renfang Chen, Tao Liu, Yiming Jiang

**Affiliations:** ^1^Department of Infection Medicine, Wuxi No. 5 People's Hospital, Wuxi 214000, China; ^2^Department of Integrated Traditional Chinese and Western Medicine, Nanjing University of Chinese Medicine, Nanjing 210000, China; ^3^Oncology Research Institute, The Fourth People's Hospital Affiliated to Jiangnan University, Wuxi 214000, China

## Abstract

**Background:**

*Herpes simplex virus* type 2 (HSV-2) infestation was the most widespread STD (sexually transmitted diseases) among humans and was the leading cause of infectious recurrent genital herpes. Existing therapies against HSV-2 did incompletely restrain the comeback of activated HSV-2 infestation. *Geranium wilfordii Maxim* had long been used as traditional Chinese medicine for treating the diseases owing to its anti-inflammatory and antiviral effects. Herein, the study was designed to investigate the antiviral activity of *G.wilfordii* and its potential effect in regulating the host's immune response.

**Methods:**

To identify the stage of infection at which the compounds inhibited HSV-2, we performed virucidal, therapeutic, and prophylactic assays. The antiviral efficacy was evaluated by the analysis of viral components HSV-2 *gD* and *VP16*. The antiviral activities of these compounds were also evaluated by phenotypic analysis, such as cell proliferation and apoptosis. Molecular docking studies on candidate compounds were done to indicate binding interactions between the compounds and adopted compound targets.

**Results:**

Quercetin, corilagin, and geraniin inhibited the replication of HSV-2, with geraniin showing greater TI. The obtained IC_50_ value of quercetin was 204.7 *μ*M and TI (IC_50_/EC_50_) was 5.1, whereas the obtained IC_50_ value of corilagin was 118.0 *μ*g/ml and TI was 4.05. Geraniin exhibited prominent antiviral activity with an IC_50_ of 212.4 *μ*M and an EC_50_ of 18.37 *μ*M, resulting in a therapeutic index (TI) of 11.56. Geraniin showed important *in vitro* virucidal activity through blocking viral attachment. Compared with the virus group, the apoptosis rates in quercetin-, corilagin-, and geraniin-treated groups were significantly decreased (*p* < 0.001).The expressions at the transcription genes of virus own replication key factors (including HSV-2 *gD* and *VP16*) and cytokines (including *TBK1*) of infected cells treated with quercetin, corilagin, and geraniin were inhibited. The *in silico* approaches demonstrated a high number of potential strong intermolecular interactions as hydrogen bonds between geraniin, corilagin, and the activity site of HSV-2 *gD*. Molecular docking studies demonstrated the effects of corilagin by targeting *TBK1*.

**Conclusions:**

Together, these results highlighted the importance of *G.wilfordii* treatment in HSV-2 infection and underscored its therapeutic potential. However, additional *in vitro* and *in vivo* research was required to validate our findings.

## 1. Introduction

HSV-2 represented as a commonest contributor to genital ulcer illness globally, with epidemiological researches unanimously showing a tight association for HSV-2 and the threat of HIV infection and spreading [1]. HSV-2 was enveloped double-stranded DNA virus belonging to *Herpesviridae* [2]. HSV-2 was neurotropic pathogen that infected epithelial tissues and nerve termini, before retrograde spread within the peripheral nervous system, wherein viral latency was established [3]. Treatments currently directed against HSV infections were nucleoside analogs such as acyclovir, valacyclovir, penciclovir, and famciclovir that targeted viral DNA polymerase [4]. While current treatments inhibited active DNA replication during reactivation, there were currently no approved treatments targeting HSV-2 in their latent infection, reflecting the still-incomplete understanding of the mechanisms of latency [5].

Natural products had been essential sources of new drugs for infectious diseases [6, 7]. It was reported that some natural compounds had shown some degree of anti-HSV-2 activity [8, 9]. As a traditional Chinese herbal medicine, *G.wilfordii* was frequently used for its antibacterial and antiviral properties [10]. *G.wilfordii* contained a variety of polyphenols [11]. Polyphenols exhibited a significant antimicrobial activity against a wide range of microbial infections [12]. Owing to their specific characters, polyphenolic substances were already put forward as wide-spectrum antiviral potential agents. Remarkably, geraniin and corilagin were the dominating active tannins of *G.wilfordii*, with the content of 14.34 and 12.32 mg/g, respectively [10]. Our previous studies used network pharmacology to identify anti-HSV-2 targets and pathways of certain bioactive components, and quercetin had the highest degree and betweenness centrality, thereby indicating that quercetin had the most important position in the network [13]. Therefore in the current studies, we chose to carry out the design and experiments using geraniin, corilagin, and quercetin.

HaCaT cells were human keratinocytes that mimicked the cells HSV infects in vivo, and these cells could produce abundant quantities of HSV particles [14]. HaCaT keratinocytes also expressed *cGAS*, *STING*, *TBK1*, and *IRF3* to similar extents [15]. The antiviral activities and toxicity of compounds, quercetin, corilagin, and geraniin, were assessed *in vitro* using HaCaT cells.

*VP16*, when combined to host proteins, was also a powerful transducible factor for five HSV instant early genes. HSV-2 *VP16* protein was essential for lytic replication [16]. For all enveloped viruses, membrane fusion was a key early step for entering host cells and establishing infection. The binding of *glycoprotein D* with one of its receptors triggered the ability of *gB* to cause membrane fusion, and the *gD* determined the tropism of the HSV to the host cells [17]. *RSAD2* (*viperin*) was an interferon-induced product associated with the restraint of reproduction of a striking array of RNA and DNA viruses. *RSAD2* had been suggested to elicit these broad antiviral activities through interactions with a large number of functionally unrelated host and viral proteins [4]. In one previous experiment design, the co-localization of endogenous *gD* and *RSAD2* was detected during HSV infection, but most of the endogenous *RSAD2* mRNA would be degraded by *UL41* when HSV infection [18]. In the *cGAS*-*STING* pathway, *TBK1* and its upstream *STING* and downstream *IRF3* constituted the core mechanism of IFNI production [19], and HSV-2 interacted with this pathway through multiple mechanisms to produce immune escape [20]. *TBK1* played a pivotal function in interferon generation and was an important constituent of antiviral immunity [21]. We measured the intracellular HSV-2 *gD* and HSV-2 *VP16* gene expression levels to evaluate the effects of quercetin, corilagin, and geraniin on viral replication. Meanwhile, we assessed whether measuring compound-gene correlations would also be sufficient to elucidate compound targets. We tested whether the mRNA expression level of *TBK1* and *RSAD2* correlates with the potency of the compound.

Molecular docking of a compound molecule with its target molecule could provide vital information about the compound-receptor binding and affinity [22, 23]. Computer-aided compound design had become an important tool in discovering new molecules with particular pharmacological effects [24, 25]. Docosanol was a marketed HSV drug that inhibited viral fusion to the host cell. Docosanol was believed to prevent virus entry by interfering with the interaction between epithelial cell membrane receptors and HSV envelope proteins [26]. HSV-2 *gD* was an essential glycoprotein of HSV [27]. Furthermore, we used molecular docking to quantify the binding forces between three polyphenolic compounds and HSV-2 *gD* and compared it with that of docosanol and HSV-2 *gD*. Quercetin had interesting protective effects due to its large spectrum of biological activities. Studies had also shown that quercetin had antiviral effects on both RNA and DNA viruses [28]. To elucidate the mechanisms underlying the broad-spectrum antiviral activity of quercetin, we examined whether quercetin could interact with *RSAD2* through molecular docking. BX795 was the first *TBK1* inhibitor to be patented. One study demonstrated the antiviral activity of a kinase inhibitor, BX795, in inhibiting HSV infection [29]. This suggested that *TBK1* and its functional interactors could be promising therapeutic targets towards HSV infection. To elucidate the mechanisms underlying the anti-HSV activity of quercetin and corilagin, we used molecular docking to quantify the binding forces between three polyphenolic compounds and *TBK1* and compared it with that of MRT67307, a modified version of BX795 and *TBK1*.

Thus, the aim of the present study was to evaluate the antiherpes effects of quercetin, corilagin, and geraniin as well as investigate a potential mechanism of anti-HSV-2 action *in vitro* through a series of laboratory assays. Additionally, the candidate compounds were also assessed by molecular docking for determining the potential of physical interactions between the compounds and potential targets.

## 2. Materials and Methods

### 2.1. Cell Culture and Virus Production

The virus strains used were aciclovir-sensitive HSV-2 G strains which were kindly donated by Prof Qinxue Hu, China Institute of Virology, Wuhan, China. HSV-2 were produced by propagating virus in Vero cells, and the titers of the virus based on TCID_50_ were determined on HaCaT cells [14]. The cells were collected when 80% CPE (cytopathic effect) was observed, and HSV-2 was harvested by freezing at −80°C and thawing at 37°C and repeating it for 3 times [30]. Vero E6 cell line was donated by Qinxue Hu research group, and HaCaT cell line was obtained through Keygen Biotechnology Co., Ltd, Nanjing, Jiangsu, China. Cells were cultivated in DMEM complemented with 10% FBS and sustained in a 5% CO_2_ humidified incubator at 37°C.

### 2.2. Compound Preparation

Based on the results obtained from the reported literatures and TCMSP (traditional Chinese medicine systems pharmacology database and analysis platform) database (old.tcmsp-e.com/index.php), we decided to perform subsequent experiments using polyphenol components quercetin, geraniin, and corilagin in *G.wilfordii*. Compound structures were derived by PubChem Chemicals Database (pubchem.ncbi.nlm.nih.gov). Geraniin, corilagin, and quercetin were purchased from Chengdu Pu Fei De Biotechnology Co. Aciclovir was purchased from Sinopharm Rongsheng Pharmaceutical Co. and used as positive control.

### 2.3. MTT Assay

MTT solution was prepared by dissolving MTT powder in sterile PBS at the concentration of 5 mg/ml. After treating cells under indicated conditions, 20 *μ*l MTT liquid was placed in the cells and the cells were cultivated for another 4 hours. The MTT solvent was then withdrawn and MTT formazan was solubilized in 150 *μ*L DMSO. Absorbance was measured at OD 492 nm. Cell survival rate was defined as cell viability = [(A experimental − A background)/(A control − A background)] × 100% [31].

### 2.4. Virus Titer Determination

Viral titers were determined by TCID_50_ assay and MTT assay using HaCaT cells, which were loaded into 96-well tissue culture plates at a scale of 5,000 cells per well in DMEM and grown overnight in a monolayer to confluence. HaCaT cells were transfected with 10-fold serial dilutions of HSV-2 virus in a total final size of 100 *μ*l and cultivated at 37°C for 2 hours. Cells were then cleaned with PBS to eliminate extracellular viruses and incubated in fresh medium for 72 hours. Cell survival rate was identified by MTT assay [14].

### 2.5. Optimal Virus Infection Conditions

The same number of cells (0.5 × 10^4^) of each group were seeded into each well of a 96-well tissue culture plate, and then seeded cells were cultivated overnight in an incubator at 37°C with 5% CO_2_. After a 2-h incubation with different concentrations (5x to 100x dilution) of virus stocks, plates were washed once with PBS. And then cells were incubated with fresh DMEM for 72 hours. Cell survival rate was determined by the MTT test. After incubating for 2 min to 120 min with 30x dilution of HSV-2 stocks (TCID_50_ = 10^–1.5^), cells were washed once with PBS to clear nonbinding virus. And again the cells were incubated with fresh DMEM for 72 hours, and, subsequently, cell survival rate was determined by the MTT test [32].

### 2.6. Compound Cytotoxicity

The cytotoxicity of acyclovir, quercetin, corilagin, and geraniin were determined by MTT assay. HaCaT cells were seeded in 96-well plates and cultured in 10% DMEM for 24 h at 37°C in an atmosphere containing 5% CO_2_. The medium was then removed and working solution of the aforementioned compounds were severally added to individual HaCaT cells in plates with 6 wells in parallel for each dose and the plates were incubated for 72 h. Cells treated without the experimental compounds were used as a control. Thereafter, cell survival rate was determined by the MTT test as previously described. Subsequently, the half-maximal inhibitory concentration (IC_50_) of experimental compound solution on HaCaT cells was automatically calculated using Bliss Principle according to the cell viability values obtained above [33].

### 2.7. Optimal antiviral Concentration of Compounds

To evaluate the optimal antiviral concentration of the compounds for *in vitro* experiments, the cell survival rate was determined by MTT test. The different initial concentrations of compound dilutions were chosen according to previous literature and our preliminary experiment. Acyclovir, quercetin, corilagin, and geraniin were diluted with DMEM from 5000 ng/ml, 100 *μ*M, 100 *μ*g/ml, and 200 *μ*M, respectively, at 2-fold multiplier for altogether 10 dilutions. Both untreated cell groups and virus-infected groups were set up. Virus infection conditions were performed as previously described. After virus infection of cells for 2 h, the cells were eluted with PBS and then each group was incubated using varied dilutions together by 72 hours followed by MTT experiments as described previously [34]. Viral inhibition rates were calculated according to the following formula: (OD experiment − OD virus)/(OD control − OD virus) ×100%. The concentration of each treatment, which reduced the virus replication rates by 50% (EC_50_), was calculated using nonlinear regression in GraphPad Prism Software.

### 2.8. Anti-HSV-2 Efficacy of Compounds at Different Concentrations and in Different Modes

Virucidal assay: The direct viral inactivation was measured by MTT assay when compared to untreated controls. Mixtures of equal volumes of the ACV, quercetin, corilagin, or geraniin and 30-fold dilution of virus stock solution in serum-free DMEM were co-incubated for 120 min at 37°C. Then, confluent monolayers of HaCaT cells received these treatments and were incubated for 120 min. The supernatants were subsequently removed, and infected cells were washed once with PBS and overlaid with serum-free DMEM, followed by incubation at 72 h. Acyclovir (156.25 ng/ml) was used as a positive control in all experiments. And then cell viability was determined by MTT assays. Viral inhibition rates were calculated as described above [35].

Therapeutic assay: Confluent HaCaT cell monolayers were infected with 30-fold dilution of virus stock solution in serum-free DMEM for 120 min at 37°C. The viruses were removed after virus adsorption by washing with PBS, and cells were overlaid with DMEM containing different compound concentrations and acyclovir (156.25 ng/ml). The plates were processed after 72 h of incubation, as previously described for MTT assay. Viral inhibition rates were calculated as described above.

Prophylactic assay: Confluent HaCaT cell monolayers were pre-treated with different compound concentrations and acyclovir (156.25 ng/ml) at 37°C for 2 h before washing once with PBS. The medium was removed and 30-fold dilution of virus stock solution in serum-free DMEM was added to the cells for additional 2 h at 37°C. Unabsorbed viruses were removed by washing with PBS; cells were covered with DMEM and then processed after 72 h as previously described for the MTT assay. Viral inhibition rates were calculated as described above [36].

### 2.9. Flow Cytometry of Apoptosis

HaCaT cells were plated at 2.5 × 10^4^ cells per well in 24-well plates in DMEM overnight. HaCaT cells were then infected with HSV-2 virus as described above. Experimental design: HaCaT cells were divided into six groups; (i) the blank control group, (ii) virus-infected group without compound treatment, (iii) acyclovir (156.25 ng/ml)-treated group, (iv) quercetin (50 *μ*M)-treated group, (v) corilagin (50 *μ*g/ml)-treated group, and (vi) geraniin (100 *μ*M)-treated group. Three days after compound treatments, cell apoptosis was measured with the Annexin V apoptosis detection kit (Biyuntian Biotechnology Co. Ltd), and apoptosis data were analyzed using BD LSR II Flow Cytometer and FlowJo software [37].

### 2.10. Real-Time Fluorescence Quantitative PCR Assay

PCR primers were purchased from Invitrogen. Total RNA was extracted using TRIzol reagent (Sangon Biotech, B511311). All TaqMan expression reagents were purchased from Jiangsu realgen-bio Co. The quantitative PCR (qPCR) experiments were performed as per the manufacturer's instructions with TaKaRa RT-PCR Kit (Takara, RR064 A) [38]. All PCR primers are shown in Table 1.

### 2.11. Molecular Docking

Protein structures of HSV-2 *gD* (PDB. 4MYV), *RSAD2* (PDB : 6B4C), and *TBK1* (PDB : 4IM0) were collected from PDB database (rcsb.org). Molecule structures of acyclovir (PubChem CID 135398513), quercetin (PubChem CID 5280343), corilagin (PubChem CID 73568), geraniin (PubChem CID 3001497), docosanol (PubChem CID 12620), and MRT67307 (PubChem CID 44464263) were obtained via search from PubChem database (https://pubchem.ncbi.nlm.nih.gov).

Molecular docking was executed for accurate docking of the ligand into the protein active sites using the LibDock module in Discovery Studio (Dassault Systèmes BIOVIA, Discovery Studio Modeling Environment, Release 2017, San Diego: Dassault Systèmes, 2016) [39]. The interactions were visualized using Discovery Studio Visualizer [40]. The binding efficiency of each target to the original ligand and prototype compounds was measured using LibDock score [41]. The LibDock scores were predicted values of the free energy of protein‐ligand binding, and a higher absolute value represents a higher affinity. The most reliable docking pose of each molecule was accepted on the basis of the highest LibDock score and further appraised using Discovery studio visualizer to examine the molecular interactions.

### 2.12. Statistical Analysis

Nonlinear regression of concentration-response curves was used with GraphPad Prism 8 for determination of the IC_50_ and EC_50_ values. Statistical analyses were determined by one-way ANOVA and were considered significant when *p* < 0.05.

## 3. Results

### 3.1. The Main Components of *G.wilfordii*

**Figure 1:**
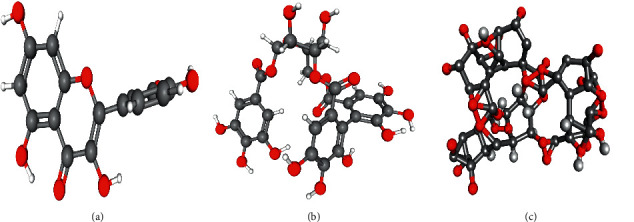
(a) 3D structures of quercetin. (b) 3D structures of corilagin. (c) 3D structures of geraniin.

Based on the results obtained from TCMSP database and PubChem database, the composition of *G.wilfordii* are presented in Table 2, while 3D structures of quercetin, corilagin, and geraniin are presented in Figure 1. Quercetin and geraniin were both relatively well absorbed in the intestine, while corilagin had poor oral bioavailability. However, the clinical application of geraniin was limited, due to its poor drug-like physicochemical properties, whereas corilagin exhibited better drug-like properties.

### 3.2. Optimal Virus Infection Conditions

**Figure 2:**
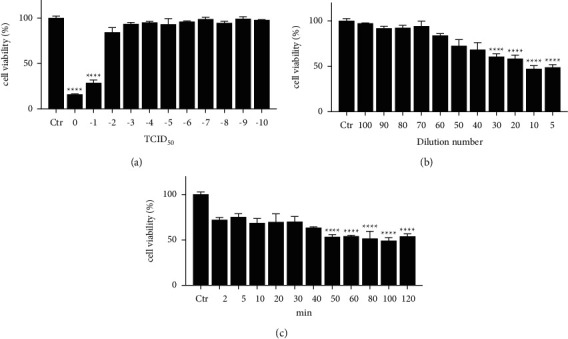
(a) TCID_50_-based virus titer assay. (b) Determination of the concentration of virus dilutions to meet the experimental conditions. (c) Determination of virus adsorption time to meet experimental conditions. ^*∗*^*p* < 0.05, ^*∗∗*^*p* < 0.01, ^*∗∗∗*^*p* < 0.001, and ^*∗∗∗∗*^*p* < 0.0001.

The TCID_50_ value of HSV-2 stocks was 10^–1.5^ (Figure 2(a)). Our results indicated that significant difference in viability was observed (*p* < 0.0001) at 5x to 30x dilution of virus stock group with less than 60% cell viability (Figure 2(b)). We observed (*p* < 0.0001) a sharp downward cell viability under the condition of virus adsorption for 50 min to 120 min with less than 60% cell viability (Figure 2(c)). In this assay, HaCaT cells were incubated with 30x dilution of virus stocks for 2 h. These conditions were employed in all experiments described below, except where specifically noted.

### 3.3. Compound Cytotoxicity

**Figure 3:**
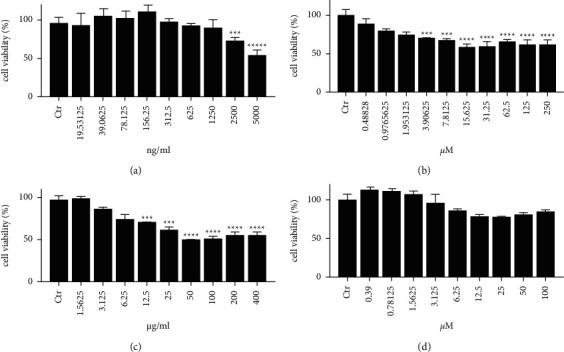
The cellular survival rates of HaCaT cells treated with acyclovir, quercetin, corilagin, and geraniin. (a) Aciclovir; (b) Quercetin; (c) Corilagin; and (d) Geraniin. ^*∗*^*p* < 0.05, ^*∗∗*^*p* < 0.01,^*∗∗∗*^*p* < 0.001, and ^*∗∗∗∗*^*p* < 0.0001.

The maximum nontoxic concentrations in HaCaT cells were defined by MTT assay. Using HaCaT cell as a prototypical cell line, strong discrimination of cytotoxicity (cell viability = 53.9%, *p* < 0.0001) was clearly apparent at 5000 ng/ml of acyclovir (Figure 3(a)). Quercetin showed significant cytotoxicity (cell viability＜60%, *p* < 0.0001) at concentrations of ≥15.625 *μ*M (Figure 3(b)). Corilagin exhibited significant cytotoxicity (cell viability＜60%) at concentrations of ≥50 *μ*g/ml (Figure 3(c)). Geraniin exhibited no obvious cytotoxicity (cell viability＞70%) at experimental concentrations (Figure 3(d)).

### 3.4. Optimal antiviral Concentration of the Compounds

**Figure 4:**
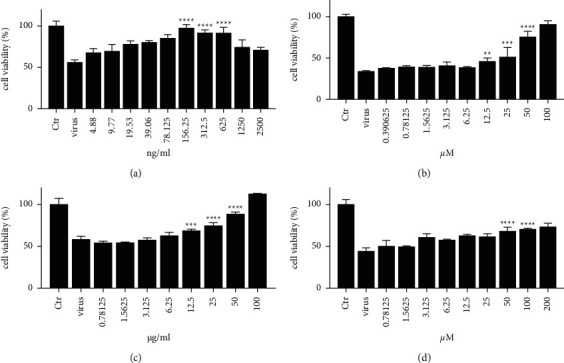
(a) Optimal antiviral concentration of acyclovir. (b) Optimal antiviral concentration of quercetin. (c) Optimal antiviral concentration of corilagin. (d) Optimal antiviral concentration of geraniin.

The prerequisite for antiviral assay was the cytotoxicity profiling of the compounds, as the cytotoxicity of the compounds was likely to impair the evaluation of the antiviral outcome of the medicine *in vitro*. The association of medication concentration and virulence might be quite distinct from the correlation of medication concentration and antiviral potency. Among the concentrations employed, 100 *μ*M of quercetin, 100 *μ*g/ml of corilagin, and 200 *μ*M of geraniin solution would partly precipitate out. As shown in Figure 4, 156.25 ng/ml of acyclovir (Figure 4(a)), 50 *μ*M of quercetin (Figure 4(b)), 50 *μ*g/ml of corilagin (Figure 4(c)), and 100 *μ*M of geraniin (Figure 4(d)) were the ones exhibiting the strongest cytoprotective effect (*p* < 0.0001). Hence, these concentrations were selected to perform the following experiments.

**Figure 5:**
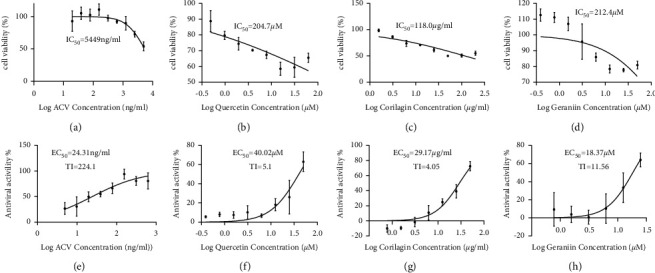
The cellular survival curves of HaCaT cells treated with acyclovir, quercetin, corilagin, and geraniin. (a) Aciclovir; (b) quercetin; (c) corilagin; and (d) geraniin. The antiviral activities of acyclovir, quercetin, corilagin, and geraniin against HSV-2. (e) Aciclovir; (f) quercetin; (g) corilagin; and (h) geraniin. IC_50_, 50% inhibitory concentration; EC_50_, 50% effective concentration; TI, therapeutic index (IC_50_/EC_50_ for anti-HSV-2); acyclovir as the positive control. ^*∗*^*p* < 0.05, ^*∗∗*^*p* < 0.01, ^*∗∗∗*^*p* < 0.001, and ^*∗∗∗∗*^*p* < 0.0001.

The inhibitory activities of acyclovir, quercetin, corilagin, and geraniin are summarized in Figure 5 along with therapeutic index (TI) values given as the ratio IC_50_/EC_50_. Quercetin, corilagin, and geraniin inhibited the replication of HSV-2, with geraniin showing greater TI. The obtained IC_50_ value of acyclovir was 5449 ng/ml (Figure 5(a)) and a therapeutic index (IC_50_/EC_50_) was 224.1 (Figure 5(e)).

The obtained IC_50_ value of quercetin was 204.7 *μ*M (Figure 5(b)) and a therapeutic index (IC_50_/EC_50_) was 5.1 (Figure 5(f))), whereas the obtained IC_50_ value of corilagin was 118.0 *μ*g/ml (Figure 5(c))) and a therapeutic index (IC_50_/EC_50_) was 4.05 (Figure 5(g)). In addition, geraniin exhibited prominent antiviral activity with an IC_50_ of 212.4 *μ*M (Figure 5(d)) and an EC_50_ of 18.37 *μ*M, resulting in a therapeutic index (TI) of 11.56 (Figure 5(h)).

### 3.5. Viral Inhibition Rates under Different Conditions of Compound Presence

**Figure 6:**
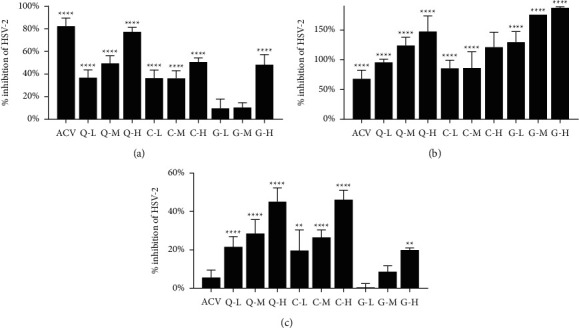
(a) Anti-HSV-2 activity of acyclovir, quercetin, corilagin, and geraniin in therapeutic assay using HaCaT cells. (b) Anti-HSV-2 activity of acyclovir, quercetin, corilagin, and geraniin in virucidal assay using HaCaT cells. (c) Anti-HSV-2 activity of acyclovir, quercetin, corilagin, and geraniin in prophylactic assay using HaCaT cells. Low (L), medium (M), and high (H) markers represented compounds with low, medium, and high doses, respectively. Quercetin doses in the low-, medium-, and high-dose groups were 12.5, 25, and 50 *μ*M, respectively. Corilagin doses in the low-, medium-, and high-dose groups were 12.5, 25 and 50 *μ*g/ml, respectively. Geraniin doses in the low-, medium-, and high-dose groups were 25, 50, and 100 *μ*M，respectively. Differences in the anti-HSV-2 activity of the samples in comparison to the viral control were analyzed by one-way ANOVA (^*∗*^*p* < 0.05, ^*∗∗*^*p* < 0.01, ^*∗∗∗*^*p* < 0.001, and ^*∗∗∗∗*^*p* < 0.0001).

All concentrations of quercetin, corilagin, and geraniin reduced HSV-2 replication in a dose-dependent manner. In therapeutic assay, acyclovir showed significant inhibition of HSV-2 with an 82.32% viral suppression rate. High-dose quercetin exhibited similar viral inhibition rates (77.286%) comparable to acyclovir (Figure 6(a)). Corilagin and geraniin showed an inferior inhibition rate at high dose (50.58 and 48.3% compared to the control, respectively). In virucidal assay, the viral inhibition rate of geraniin was 187.2% (Figure 6(b)), which proved superior to the 67.99% inhibition rate seen for acyclovir. Corilagin and quercetin showed an inferior inhibition rate at high dose (121.2 and 147.3% compared to the control, respectively). In prophylactic assay, the inhibition rate of acyclovir was markedly decreased (5.49% compared to the control). Corilagin and quercetin showed notable inhibition rates at high dose (46.03 and 45.1% compared to the control, respectively) (Figure 6(c)).

### 3.6. Flow cytometry Analysis of Apoptosis

**Figure 7:**
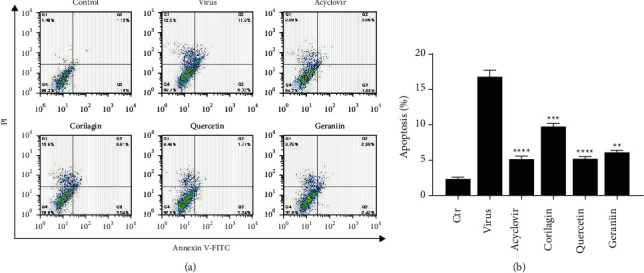
(a) The representative flow cytometry plots showed proportions of four groups' HaCaT cells after different treatments. (b) A histogram showing quercetin, corilagin, and geraniin treatment inhibited cell apoptosis. (^*∗*^*p* < 0.05, ^*∗∗*^*p* < 0.01, ^*∗∗∗*^*p* < 0.001, and ^*∗∗∗∗*^*p* < 0.0001).

To examine the compound's antiviral mechanism of action, we examined apoptosis *in vitro* through Annexin V/propidium iodide flow cytometry. As depicted in Figure 7(a), after 72 -h treatment, HSV-2-infected HaCaT cells underwent obviously late apoptosis and cell death rate (11.5% and 12.5%, respectively) during lytic infection compared to control group (1.12% and 1.45%, respectively). Compared with the virus group, the apoptosis rates in the other four compound-treated groups were significantly decreased (*p* < 0.001) (Figure 7(b))), suggesting the inhibition of cell apoptosis by acyclovir, quercetin, corilagin, and geraniin treatment. We found that HaCaT cells in quercetin-treated conditions exhibited apoptosis rates comparable to that of acyclovir (5.29% and 5.22%, respectively).

### 3.7. Quantification of *RSAD2*, *TBK1*, HSV-2 *gD*, and *VP16* under Different Modes of Action for Each Compounds

**Figure 8:**
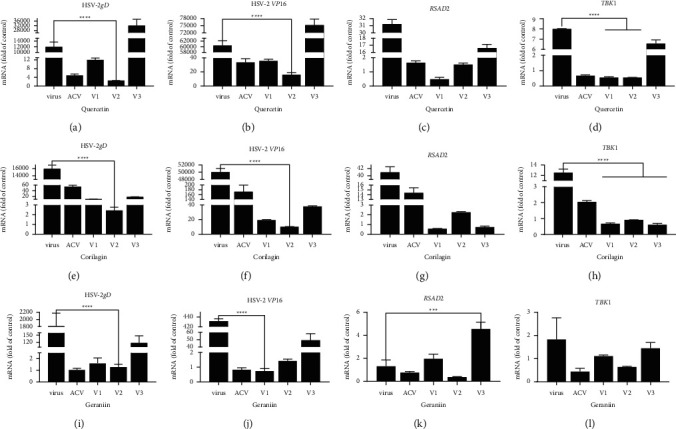
The mRNA levels of each factor in cells treated with different modes. (a)–(d) Quercetin-treated group. (e)–(h) Corilagin-treated group. (i)–(l) Geraniin-treated group. Virus meant positive control without compound treatment. ACV represented acyclovir-treated group. V1 represented virucidal assay group. V2 represented therapeutic assay group. V3 represented prophylactic assay group. ^*∗*^*p* < 0.05, ^*∗∗*^*p* < 0.01, ^*∗∗∗*^*p* < 0.001, and ^*∗∗∗∗*^*p* < 0.0001.

To determine if quercetin, corilagin, and geraniin specifically impacted virus replication, we checked viral *gD* and *VP16* levels by QPCR. Quercetin, corilagin, and geraniin showed the greatest inhibition in therapeutic assay, being less potent in virucidal assay, and least potent in prophylactic assay (Figures 8(a), 8(b), 8(e), 8(f), 8(i), and 8(j)). The action mode in prophylactic assay might affect the inhibitory effects of quercetin targeting HSV-2 *gD* and *VP16*. The inhibitory effect in virucidal assay was commensurable to that of geraniin in therapeutic assay (Figure 8(i), 8(j)). We inferred from this that geraniin might have the potential to function as antiviral drugs by directly interacting with critical proteins of the virus.

The ribonuclease UL41 of HSV could degrade the mRNA of *RSAD2* to promote HSV replication [43]. Vaginal tissue from mice infected locally with HSV-2 showed strong *RSAD2* expression in cells located to the area of infection [44]. The action of geraniin in prophylactic assay promoted the expression of interferon (IFN)-stimulated genes (ISGs) *RSAD2* compared to virus-infected group (*p* < 0.001) (Figure 8 K).

*TBK1* was an important regulator of innate immune responses. Optimized *TBK1* action was indispensable for virus clearance, and overexcited *TBK1* activation might accelerate inflammatory damage during viral infection [21]. BX795, an antagonist for *TBK1*, vigorously restrained HSV-1 infection by inhibiting viral protein synthesis. Quercetin and corilagin showed inhibitory effects on *TBK1* indicating that they might inhibit HSV-2 replication by targeting *TBK1* (Figure 8(d), 8(h))).

### 3.8. Molecular Docking Studies

**Figure 9:**
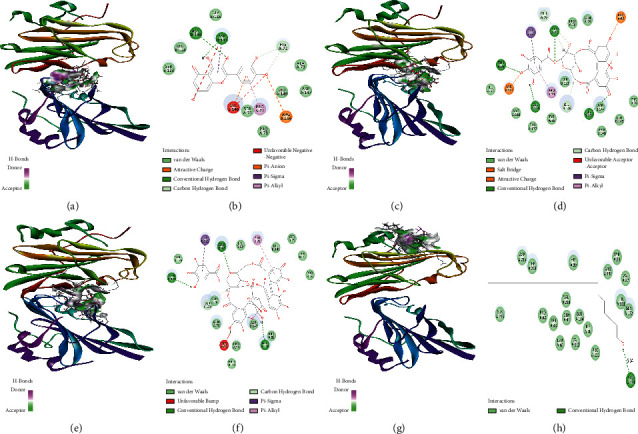
Interactions observed between the ligand molecules and the binding pocket of the HSV-2 *gD* visualized by Discovery studio. Green colour represented the hydrogen bond interaction between the target and the ligand molecules. (a) 3D interaction pattern of HSV-2 *gD*-quercetin complex. (b) 2D pattern of HSV-2 *gD*-quercetin complex. (c) 3D interaction pattern of HSV-2 *gD*-corilagin complex. (d) 2D pattern of HSV-2 *gD*-corilagin complex. (e) 3D interaction pattern of HSV-2 *gD*-geraniin complex. (f) 2D pattern of HSV-2 *gD*-geraniin complex. (g) 3D interaction pattern of HSV-2 *gD*-docosanol complex. (h) 2D pattern of HSV-2 *gD*-docosanol complex.

**Figure 10:**
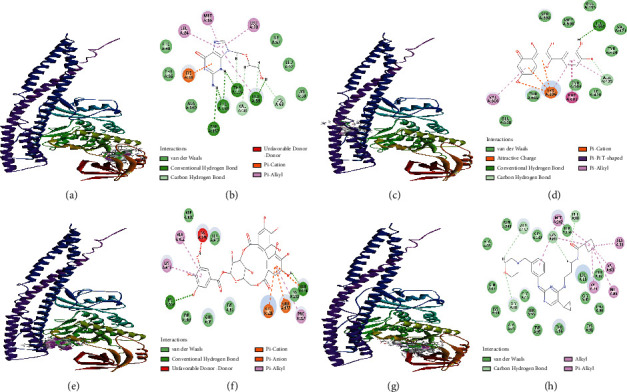
Interactions observed between the ligand molecules and the binding pocket of the *TBK1* visualized by Discovery studio. (a) 3D interaction pattern of *TBK1*-aciclovir complex. (b) 2D pattern of *TBK1*-aciclovir complex. (c) 3D interaction pattern of *TBK1*-quercetin complex. (d) 2D pattern of *TBK1*-quercetin complex. (e) 3D interaction pattern of *TBK1*-corilagin complex. (f) 2D pattern of *TBK1*-corilagin complex. (g) 3D interaction pattern of *TBK1*-MRT67307 complex. (h) 2D pattern of *TBK1*-MRT67307 complex.

We speculated that quercetin, corilagin, and geraniin activity could fulfill a specific regulatory function by acting on particular targets including viral components and immune response-related factors. As a preliminary test for this hypothesis, we conducted molecular docking of quercetin, corilagin, and geraniin targeting HSV-2 *gD*, *RSAD2*, and *TBK1* proteins (PDB ID : 4MYV, 6B4C, and 4IM0, respectively). The interaction energies of the quercetin, corilagin, and geraniin with HSV-2 *gD*, *RSAD2*, and *TBK1* proteins are shown in Table 3. Docosanol could repress HSV-2 entry by disrupting the mutual association for epithelial cell layer receptors and HSV-2 envelope proteins. BX795 could inhibit the phosphorylation of *TBK1* and block HSV infection. MRT67307, a modified version of BX795, was a potent *TBK1* inhibitor. The compounds docosanol (LibDock score = 101.728) (Figure 9(g) and 9(h))and MRT67307 (LibDock score = 119.478) (Figure 10(g) and 10(h))were selected on the basis of the best interaction energies for further analysis.

**Figure 11:**
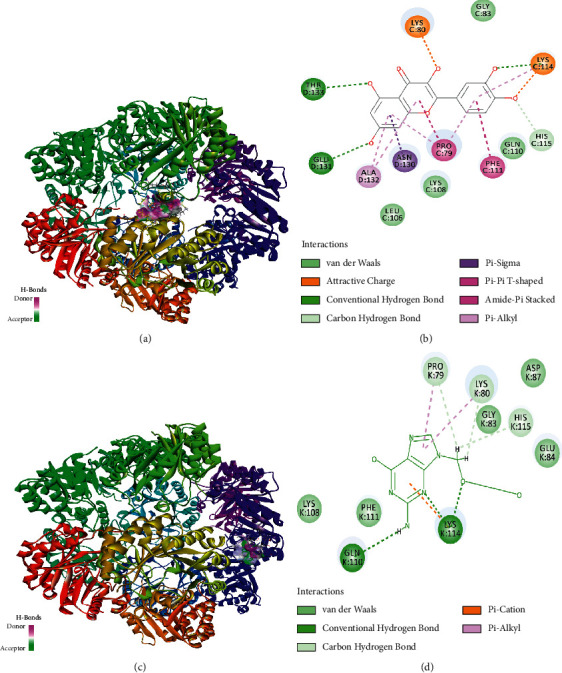
Interactions observed between the ligand molecules and the binding pocket of the *RSAD2* visualized by Discovery studio. (a) 3D interaction pattern of *RSAD2*-quercetin complex. (b) 2D pattern of *RSAD2*-quercetin complex. (c) 3D interaction pattern of *RSAD2*-aciclovir complex. (d) 2D pattern of *RSAD2*-aciclovir complex.

The binding modes of HSV-2 *gD* with four selected compounds are displayed in Figure 9. Geraniin demonstrated high occupation in the active site of HSV-2 *gD* (Figure 9(e), 9(f)). In addition, the conformation of quercetin, corilagin, and geraniin reviewed by the docking simulations occupied the same pocket with numerous hydrogen bonds for HSV-2 *gD* (Figure 9(a), 9(c), 9(e)). Geraniin interacted with HSV-2 *gD* at site 3 by twelve Van der Waals, one carbon-hydrogen, and three hydrogen bonds (Figure 9(f)). Corilagin interacted with HSV-2 *gD* at site 3 by eleven Van der Waals, two carbon-hydrogen, and four hydrogen bonds (Figure 9(d))). It also formed two hydrogen bonds and six Van der Waals interactions with *TBK1* at site 2 (Figure 10(e), 10(f)). Quercetin interacted with HSV-2 *gD* at site 3 by eight Van der Waals, one carbon-hydrogen, and two hydrogen bonds (Figure 9(b)). It also formed two hydrogen bonds, four Van der Waals interactions, and one carbon-hydrogen with *RSAD2* at site 4 (Figure 11(a), 11(b)).

The docking results in Figure 9 showed that docosanol was docked into active sites of HSV-2 *gD* domain at ARG130 (Figure 9(h)), and LibDock score was 101.728. Both corilagin and geraniin could bind to HSV-2 *gD* against the residues ARG82 and GLU146 and form hydrogen bonds (Figure 9(d), 9(f)) with higher LibDock score of 139.12 and 133.906 compared with docosanol. The docking results demonstrated that quercetin was docked into active sites of *RSAD2* domain at LYS80, LYS114, HIS115, GLU131, and THR133 (Figure 11(b)) and showed good LibDock interaction energy (LibDock score = 94.3065) higher than ACV (LibDock score = 86.3175).

Structure-based molecular docking simulations in Figure 10 indicated that corilagin could bind to the active site GLU178, ARG228, and LYS567 of *TBK1* by forming hydrogen bonds (Figure 10(f)) exhibiting comparable binding scores (LibDock score = 105.018) to MRT67307 (LibDock score = 119.478). All the bonds were visible in the 3D and 2D diagram, also depicting the hydrophobic surface interaction between ligand and the receptor.

## 4. Discussion

Vero cells were usually used to propagate and grow large batches of HSV-2 for research and especially virus batches grown for use in vaccine challenge studies [45]. Although Vero cells was a model cell line for pathogenic HSV-2, they likely did not fully recapitulate all aspects of infection in primary cells, such as human genital epithelial cells, nor does this system fully recapitulate the complex cellular milieu in a human patient [46]. HaCaT culture could artificially mimic the HSV-2 infection within the real reproductive tract microenvironment [47]. In the present study, we used HaCaT cells as an *in vitro* assay platform, which was a long-lived, spontaneously immortalized human keratinocyte line with potentially different signalling pathways compared to non‐immortalized cells [15]. Our results showed that Hacat cells could be infected with HSV-2, and the infection efficiency on the HaCaT cells estimated that HaCaT cells could be used as a susceptible cell line for HSV-2 infection.

HSV-2 infection represented a serious public threat and the unavailability of potential antiviral drugs emphasized the need for identification of new leads [48]. Multiple bioassays were used to identify quercetin, corilagin, and geraniin obtained from *G.wilfordii* with promising anti-HSV-2 effects. In the three ways of therapeutic, virucidal, and prophylactic modes, quercetin, corilagin, and geraniin could exert an individual antiviral activity on the infected HSV-2 cells. By confirming the effectiveness of geraniin, one promising compound, as a virus-host cell fusion inhibitor, HSV-2 entry host cells can be prevented to treat HSV-2 infection. In addition, geraniin had lower cytotoxicity against HaCaT cells and a better therapeutic index against HSV-2. These findings strongly suggested that geraniin might be a promising candidate compound for the effective treatment of HSV-2 infection.

The function of the HSV tegument protein *VP16* played a crucial role in the HSV life cycle, and it was a powerful transcriptional activator that specifically acted on IE (Immediate Early) genes [49].

Thus, the analysis of the interaction between *VP16* and compounds would be meaningful for the further development of antiviral compounds. HSV-2 was an enveloped DNA virus. For all enveloped viruses, membrane fusion was a key early step for entering host cells and establishing infection. The binding of glycoprotein *D* with one of its receptors triggered the ability of *gB* to cause membrane fusion, and the *gD* determined the tropism of the HSV to the host cells. The inhibition of the membrane fusion process was a promising strategy for combating infection [50]. It was found by QPCR assay that quercetin, corilagin, and geraniin could attenuate the expression of HSV-2 *gD* and *VP16* at the transcription gene level. According to the above comprehensive data, the antiviral activity of quercetin, corilagin, and geraniin on infected cells was closely related to the replication process of HSV-2.

HSV-encoded molecules also interfered with cellular apoptosis [51], which was an important innate immune mechanism for eliminating pathogen-infected cells. HSV initiated apoptosis through a parallel pathway involving *cGAS* and *STING* [52]. HSV immediate-early protein *ICP0* triggered apoptosis during HSV infection to influence viral pathogenesis [53]. However, the exact mechanisms underlying the promotive effect of apoptosis on such HSV infection-associated diseases remained unknown. The detection of apoptosis had provided the tools for drug discovery as well as the experimental means for validation of drug action in clinical specimens [54]. Our *in vitro* study indicated that the rate of apoptosis was obviously higher in virally infected group compared with quercetin, corilagin, and geraniin treatment group. After treatment of HaCaT cells with quercetin, corilagin, and geraniin, the apoptosis caused by HSV-2 was inhibited, indicating that above compounds could inhibit the apoptosis of HSV-2-infected cells and this might be an important mechanism by which above compounds exerted anti-HSV-2 effects.

Molecular docking approaches had rendered perceptions into the ligand-receptor interaction manner and in the discovery of potential HSV-2 inhibitors. The availability of an inhibitor bound protein structure rendered an outstanding opportunity to provide the data linked to their interaction [55]. It would be beneficial to consolidate the derived data in structure-based drug design [56]. *TBK1* and *RSAD2* protein played an essential role in innate immunity against HSV-2 infection, meanwhile HSV-2 *gD* had prime function in the entry of virus into the host, and thus they were chosen as potential targets for antiviral search. Quercetin, corilagin, and geraniin from *G.wilfordii* were docked on the predicted binding site. Corilagin and geraniin presented the most promising binding energies targeting HSV-2 *gD*. We also compared these results with the binding potential of docosanol. The comparative analysis suggested that corilagin and geraniin showed more promising potential than anti-HSV-2 compounds docosanol. From molecular docking results based on the cumulative effect of estimated affinity and ligand efficiency, we selected corilagin, which gave similar results to the *TBK1* inhibitor MRT67307 and could be considered to act as specific antagonists. Additionally, these *in silico* and *in vitro* findings required further experimental validation to advance in the drug discovery pipeline [57].

## 5. Conclusions

There was a growing interest in naturally derived products providing outstanding health merits with relatively safe profiles, useful for the treatment of HSV-2 infections. In this article, the antiviral activity and mechanism of quercetin, corilagin, and geraniin against HSV-2 were investigated *in vitro*. Geraniin exhibited prominent antiviral activity with an IC_50_ of 212.4 *μ*M and an EC_50_ of 18.37 *μ*M, resulting in a therapeutic index (TI) of 11.56. Both quercetin and corilagin solution could provide antiviral effects for therapeutic, virucidal, and prophylactic inactivation of HSV-2 infection, and geraniin showed important *in vitro* virucidal activity, and this indicated that geraniin might exert a good anti-HSV-2 activity *in vitro* through inhibiting membrane fusion of HSV-2 targeting HSV-2 *gD*. At the level of virus replication, quercetin, corilagin, and geraniin could significantly inhibit the transcriptional gene expressions levels of HSV-2 *VP16* and *gD*. Apoptosis assay showed that the apoptosis rates of HaCaT cells infected by compound-treated HSV-2 were decreased markedly.

HSV-2 *gD* was a promising target for developing novel HSV-2 inhibitors. In this analysis, we selected a set of three compounds from *G.wilfordii* to examine their interaction posed inside the active site of the HSV-2 *gD* complex. Our findings revealed the efficacy of corilagin and geraniin against HSV-2 *gD* complex. In selected corilagin and geraniin, LYS122, ARG82, and GLU146 had the highest contribution.

Meanwhile, we studied the antagonistic behavior of the previously known molecule MRT67307 in comparison to quercetin and corilagin, which have natural origin towards its binding site in the *TBK1*. Quercetin and corilagin molecules successfully interacted with binding site of *TBK1* in the receptor. It depicted a fairly similar binding affinity for the corilagin towards the receptor as compared to MRT67307.

As docking experiments only provided stable static binding modes for a ligand inside the active site of a protein [58], further research focused on the authentication of its activity along with pharmacokinetic and pharmacodynamic properties *in vivo* and in clinical trials is required [22].

## Figures and Tables

**Table 1:** Primer sequence.

Gene	Primer sequence
*gD*	
Forward primer	CCAAATACGCCTTAGCAGACC
Reverse primer	CACAGTGATCGGGATGCTGG

*VP16*	
Forward primer	AATGTGGTTTAGCTCCCGCA
Reverse primer	CCAGTTGGCGTGTCTGTTTC

*RSAD2*	
Forward primer	TGGGTGCTTACACCTGCTG
Reverse primer	GAAGTGATAGTTGACGCTGGTT

*TBK1*	
Forward primer	TGGGTGGAATGAATCATCTACGA
Reverse primer	GCTGCACCAAAATCTGTGAGT

*GAPDH*	
Forward primer	GAGTCAACGGATTTGGTCGT
Reverse primer	GACAAGCTTCCCGTTCTCAG

**Table 2:** Active ingredients and ADME parameters of *Geranium wilfordii* Maxim.

NO.	Molecule ID	Molecule name	Chemical formula	MW	OB (%)	DL
1	MOL001002	Ellagic acid	C_14_H_6_O_8_	302.2	43.06	0.43
2	MOL000359	Sitosterol	C_29_H_50_O	414.79	36.91	0.75
3	MOL000422	Kaempferol	C_15_H_10_O_6_	286.25	41.88	0.24
4	MOL005067	Furosin	C_27_H_22_O_19_	650.49	40.53	0.29
5	MOL005073	Ethyl Brevifolincarboxylate	C_15_H_12_O_8_	320.27	30.86	0.33

6	MOL000006	Luteolin	C_15_H_10_O_6_	286.25	36.16	0.25
7	MOL000098	Quercetin	C_15_H_10_O_7_	302.25	46.43	0.28
8	MOL005064	Dehydrogeraniin	C_41_H_28_O_28_	968.68	59.57	0.01
9	MOL005079	Corilagin	C_27_H_22_O_18_	634.49	3.01	0.44

ADME [42], absorption, distribution, metabolism, and excretion; OB, oral bioavailability; DL, drug-like properties.

**Table 3:** Docking scores of experimental compounds with potential targets.

Targets	Compound	Close contact residues	Amino acid involved in hydrogen bond	LibDock score
*gD*	Quercetin	PRO74, ARG166, GLU146	HIS72, ASP147, ASN148	84.8417
*gD*	Corilagin	PRO74, LYS122, VAL126	TTHR56, SER75, GLU76, ARG82 GLU175, LEU124, GLU146	139.12
*gD*	Geraniin	PRO78, LYS122	ARG82, ASN121，SER123, GLU146	133.906
*gD*	Docosanol	—	ARG130	101.728
*RSAD2*	Quercetin	PRO227, ALA412, LYS416, GLU177	LYS80, LYS114, HIS115, GLU131, THR133	94.3065
*RSAD2*	Aciclovir	—	PRO79, LYS80, GLN110, LYS114, HIS115	86.3175
*TBK1*	Corilagin	PRO227, ALA412, LYS416, GLU177	GLU178, ARG228, LYS567	105.018
*TBK1*	Aciclovir	MET86, LEU70, LEU84	LYS38, LEU59, VAL68, LYS69 THR156, ASP157, PHE158	98.4021
*TBK1*	Quercetin	LYS396, PHE601, VAL606	ILE397, ALA425	81.9411
*TBK1*	MRT67307	VAL23, VAL68, MET86, MET142, ALA36	GLY18, PHE88, CYS89, ASP157	119.478

## Data Availability

All data are available from the corresponding author upon reasonable request.
